# Supplementary material for: A medullary centre for lapping in mice
Source: Nat Commun. 2021 Nov 2;12:6307. doi: 10.1038/s41467-021-26275-y (PMC8563905; doi:10.1038/s41467-021-26275-y)
Supplement: Supplementary file 1 — Supplementary Information [file 41467_2021_26275_MOESM1_ESM.pdf]

Supplementary figures

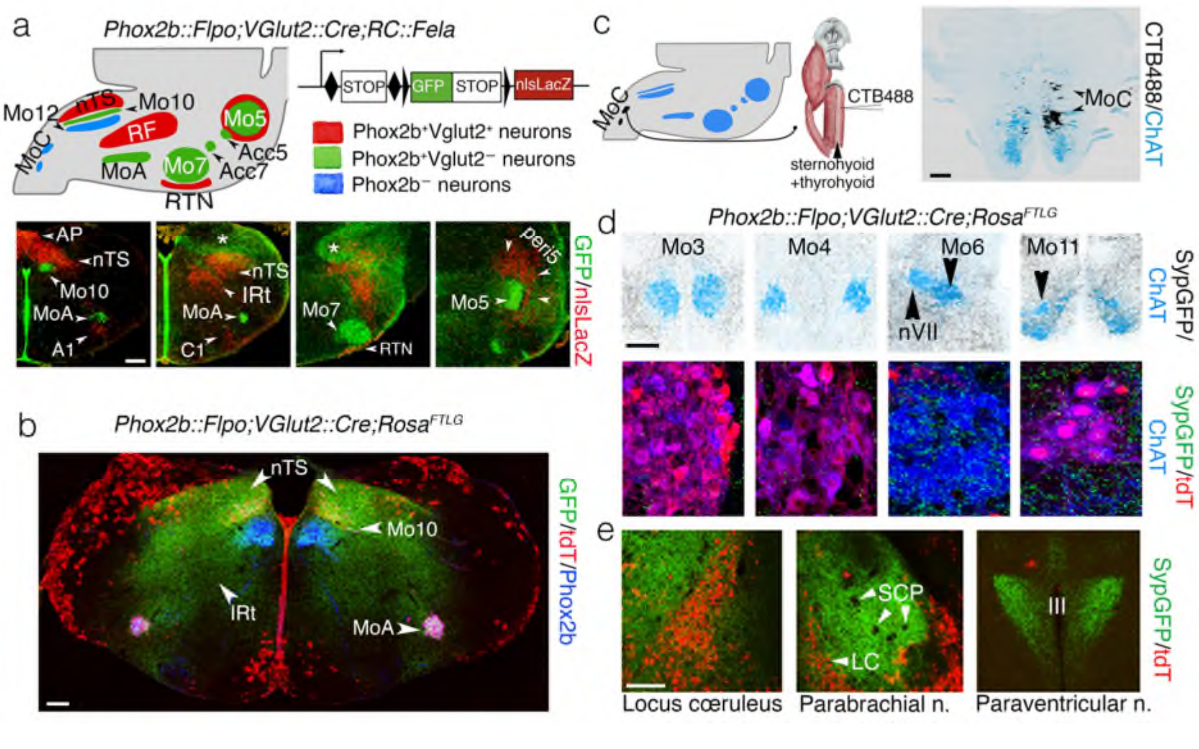

Supplementary Fig. 1. (a) (Upper) Strategy for transgenic labeling of glutamatergic *Phox2b*$^{+}$ interneurons using the *FELA* transgene, and summary of the results. (Lower) Coronal sections through the pons and medulla of a *vGlut2::Cre;Phox2b::Flpo;RC::Fela* embryo at E17.5. Branchiomotor and visceromotor neurons (cholinergic and Phox2b^{+}) express GFP (green), while interneurons that are glutamatergic and Phox2b^{+} express *nlsLacZ* (red). The dorsal aspect of the hindbrain harbors numerous *GFP*$^{+}$ astrocytes (asterisks), likely born from Phox2b^{+} progenitors, in p3/pMNv (*12*)(*13*) or possibly dB2. (**b**) Coronal section through the medulla of a *vGlut2::Cre;Phox2b::Flpo;Rosa*^{FTLG} embryo at E17.5, showing that branchial and visceral neurons have retained tdT expression (since they undergo recombination by *Flpo* alone), while glutamatergic *Phox2b*$^{+}$ neurons in the nTS or IRt have lost tdT, to gain *Syp-GFP* expression after dual *Cre* and *Flpo* recombination. *Syp-GFP* marks fields of synaptic boutons in discrete areas of the medulla. (**c**) (left) Strategy for labeling the motor nucleus for the infrahyoid muscles (sternohyoid and thyrohyoid) (MoC), by retrograde transport of CTB. (right) transverse section at the spinal-medulla junction showing two islands of labelled motoneurons (black). **(d)** Motor nuclei for extraocular muscles (Mo3, Mo4 and Mo6) and for the trapezius and sternocleidomastoid (Mo11), do not

receive labelled boutons in a *vGglut2::Cre;Phox2b::Flpo;Rosa*^{FTLG} background at P8. (**E**) Known sites of projection from *Phox2b*$^{+}$ glutamatergic interneurons (including C1 neurons and the nTS) are covered by *GFP*$^{+}$ boutons: the parabrachial nucleus (*14*), the locus coeruleus and the paraventricular nucleus of the hypothalamus (*15*). LC, locus coeruleus; SCP, superior cerebellar peduncle; III, third ventricle. Scale bars, 200 μm.

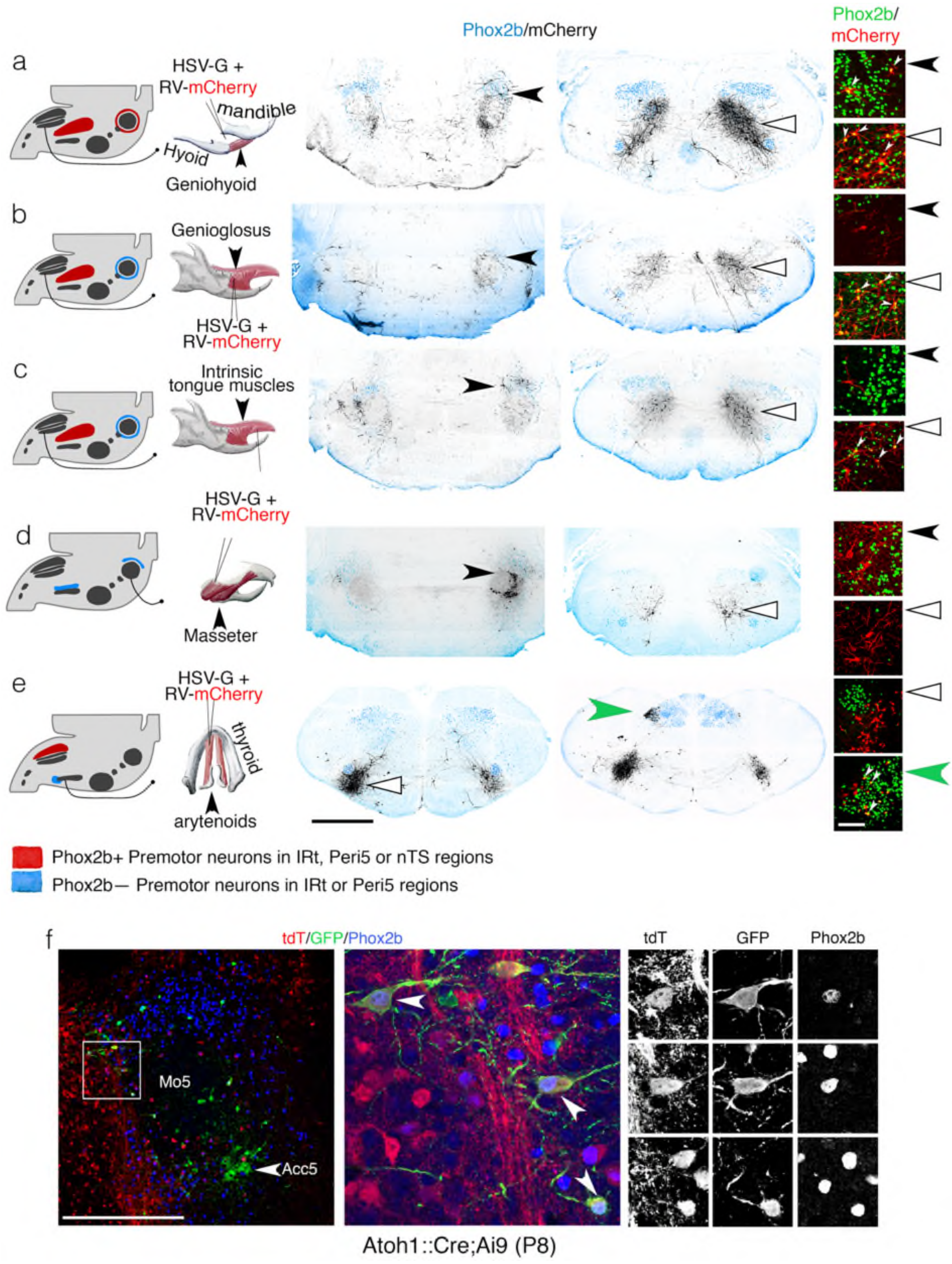

Supplementary Fig. 2. Retrograde monosynaptic labeling of the premotor neurons for the geniohyoid (**a**), genioglossus (**b**), intrinsic tongue muscles (**c**), masseter (**d**) and thyro-arytenoid (**E**). (Left column) Labeling strategy and summary of the results. (Middle column) Sections through the pons at the level of Mo5 (**a**-**d**, left), or at the level of the IRt (**a**-**d**, right, and **e**, left and right) showing the filled premotor neurons (black) on the landscape of Phox2b+ neurons (blue). (Right column) Close ups of filled premotor neurons (red) either expressing *Phox2b* (green nuclei), or not.

The thyro-arytenoid and masseter have no *Phox2b*$^{+}$ premotor neurons in the IRt or Peri5. (**f**) Coronal section through the peri5 region of a Cre-reporter *Ai9* mouse crossed with *Atoh1::Cre*, whose posterior digastric muscle was co-injected with a DG-rabies virus encoding GFP and a helper HSV-G, counter-stained for *Phox2b*, at 3 magnifications from left to right. Three triple-labeled neurons are highlighted, which thus have a history of both *Atoh1* and *Phox2b* expression, and are premotor to the posterior digastric. Scale bar, (**a**-**e**), 1 mm (**insets**, 150 μm); (**f**), 500μm.

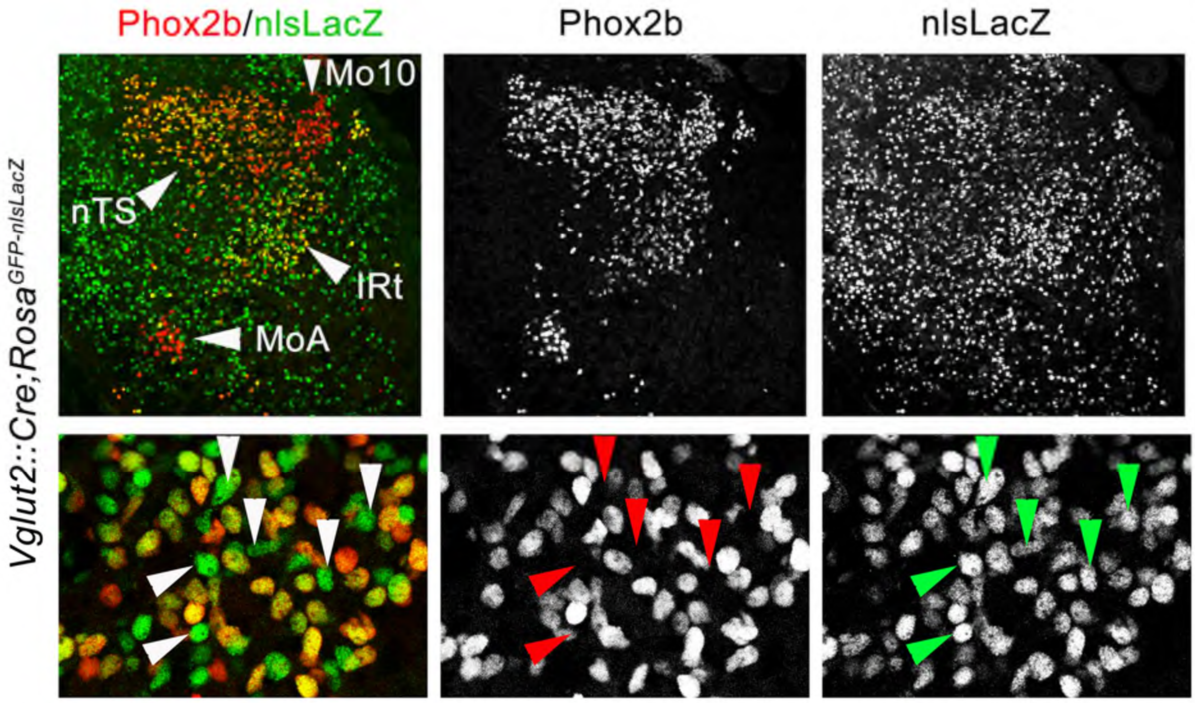

Supplementary Fig. 3. Double immunofluorescence for *Phox2b* and *nlsLacZ* in a transverse section through the hindbrain of a *Vglut2::Cre*;*Rosa*$^{GFP\text{-}nlsLacZ}$ pup at P0 showing that in IRT^{Phox2b} (shown at low magnification in upper panels — marked IRt in upper right panel — and at high magnification in lower panels) glutamatergic (*nlsLacZ*$^{+}$) *Phox2b*$^{+}$ neurons (yellow cells) are intermingled with other glutamatergic (*nlsLacZ*$^{+}$) *Phox2b*-negative neurons (green cells, marked by arrowheads in lower panels). In contrast, the overwhelming majority glutamatergic neurons in nTS are *Phox2b*$^{+}$.

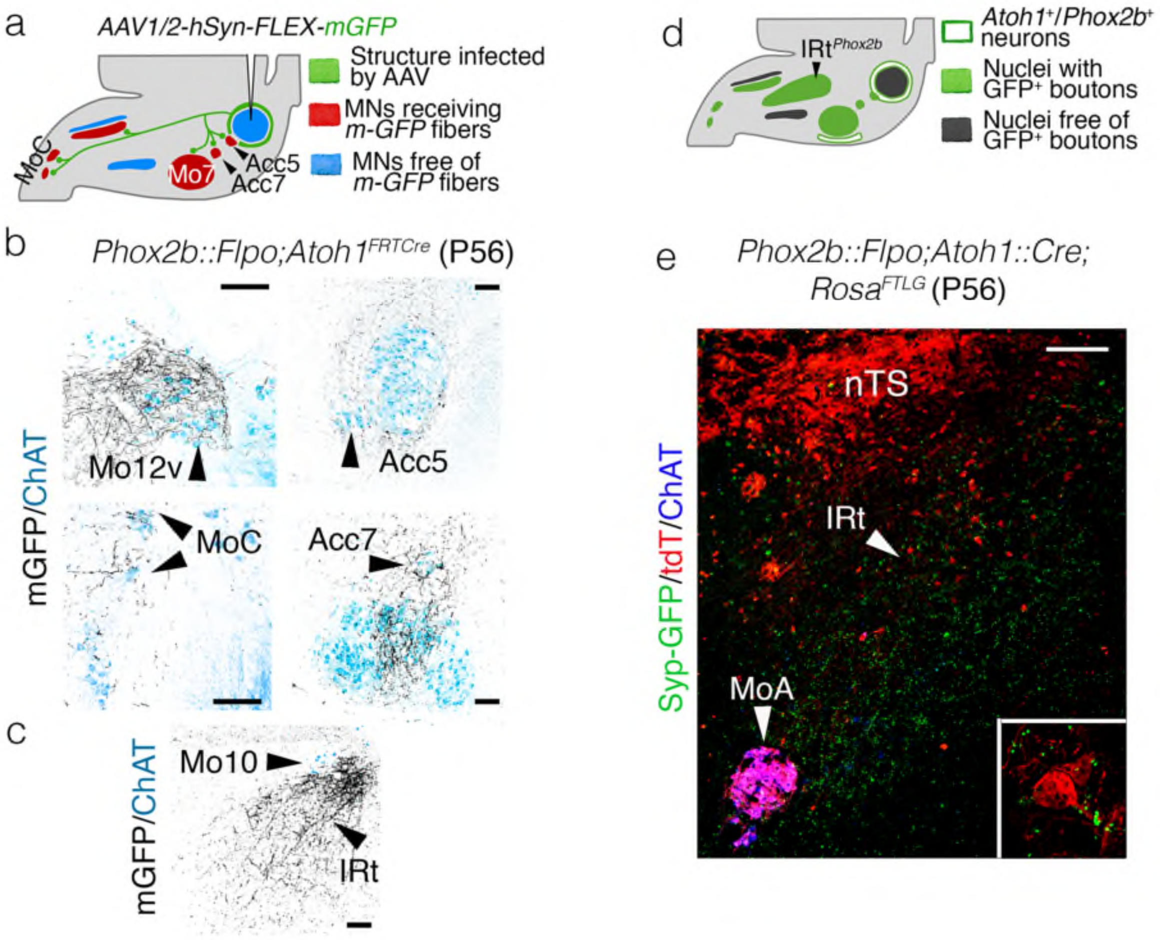

Supplementary Fig. 4. (**a**): Strategy for viral tracing the projections of Peri5^{Atoh1} and summary of the results. (**b)**: Coronal sections through the motor nuclei (ChAT^{+}, blue) that receive projections from Peri5^{Atoh1}, labelled with *mGFP* encoded by the AAV anterograde virus (black). (**c**): Coronal sections through the medulla showing *mGFP*-labeled fibers in the IRt. (**d**): Strategy for transgenic labeling projections from *Atoh1*$^{+}$/*Phox2b*$^{+}$ cells (Peri5^{Atoh1} and RTN) using the *Rosa*^{FTLG} transgene and summary of the results already visible in Fig 3A. (**e**) Projections from Peri5^{Atoh1} + RTN on IRt^{Phox2b} at intermediate and high (inset) magnifications. Scale bars (**b**-**e**) 200 µm.

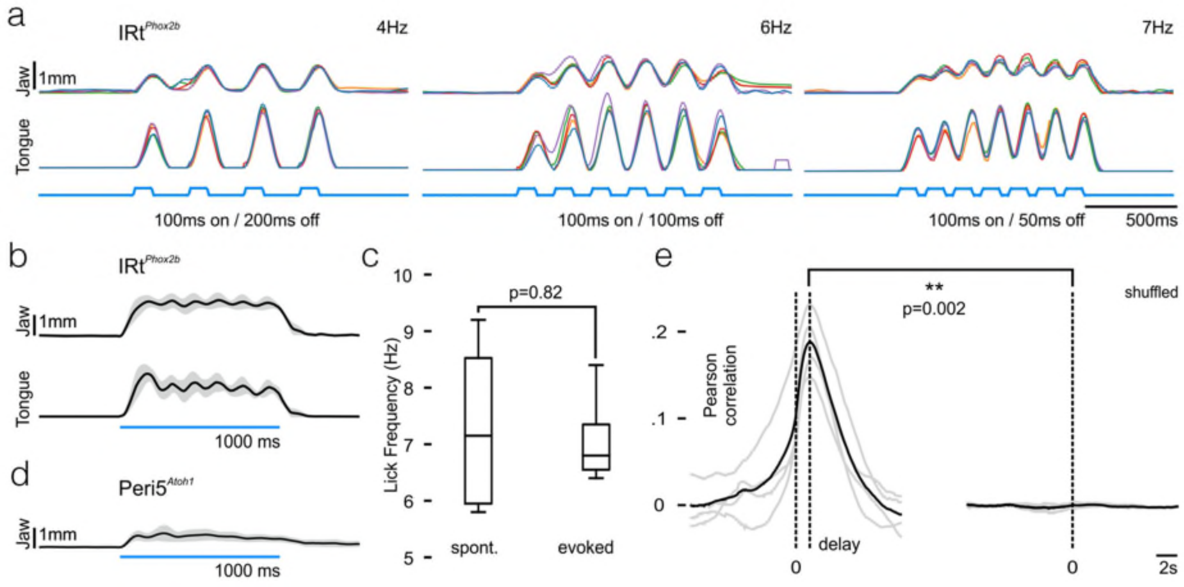

Supplementary Fig. 5. (**a**) Example of tracked jaw and tongue position on the Y axis upon stimulation of IRt^{Phox2b} with 1000ms long trains of 100ms pulses at 4Hz (33% duty cycle), 6Hz (50% duty cycle) and 7Hz (67% duty cycle) over multiple trials (n=5). (**b**) Grand average of tracked position of jaw and tongue on the Y axis upon 1000 ms stimulation of IRt^{Phox2b} (n=4 mice, 34 trials). (**c**) Box plot of spontaneous (mean=7.3 Hz ±0.8 SEM, median=7.2, Q1-Q3 =6-8.5, whiskers=9.2-5.8) (n=4 mice) and evoked licking frequency (mean=7.1 Hz ±0.4 SEM, median=6.8, Q1-Q3=6.6-7.4, whiskers=6.4-8.4) (n=4 mice). No significant difference between evoked and spontaneous frequency (p=.82, y a two-sided paired t-test, confidence interval 95%). (**d**) Grand average (n=4 mice, 21 trials) of tracked position of the tongue on the Y axis upon 1000 ms stimulation of Peri5^{Atoh1}. (**e**) (left) Mean shifted correlation curves displayed for photometry recordings of IRtPhox2b during volitional licking from individual animal (n=4, gray) and the overall mean (black), displaying a 1.3s delay between lick port contact and maximum correlation; (right) same computation on the same data but with the lick port signal shuffled, p=.002 (two-sided paired t-test, confidence interval 95%).

Supplementary Table 1. Primer sequences for genotyping and probe synthesis

Phox2b ::Flpo	Forward 5'-GA**G** CTT CGA CAT CGT GAA CA-3'<br>Reverse 5'-ACA GGG TCT TGG TCT TGG TG-3'
Phox2b ::Cre	Forward 5'-GGC CGG TCA TTT TTA TGA TC-3'<br>Reverse 5' -GAA ATC AGT GCG TTC GAA CGC TAG
Vglut2 ::Cre	Forward 5'-TGA TGG ACA TGT TCA GGG ATC-3'<br>Reverse 5'-GAA ATC AGT GCG TTC GAA CGC TAG-3'
Atoh1 ::FRTCre	Forward 5'-GCT GAT CCG GAA CCC TTA AT-3'<br>Reverse 5'-AGG AAC TGC TTC CTT CAC GA-3'
ATOH1 ::CRE	Forward 5'-TGA TGG ACA TGT TCA GGG ATC-3'<br>Reverse 5'-GAA ATC AGT GCG TTC GAA CGC TAG-3'
FoxG1-^{iresCre}	WT Forward 5'-TAG TGA AAC AGG GGC AAT GG-3'<br>Mut Forward 5'-ACC CTG CCC TGT GAG TCT T-3'<br>Reverse 5'-TTC TCC CAC ATT GCA CCT C-3'
Olig3 ::Cre^{ert2}	Forward 5'-TGA TGG ACA TGT TCA GGG ATC-3'<br>Reverse 5'-GAA ATC AGT GCG TTC GAA CGC TAG-3'
Tau :: Syp $^{-}$GFP	LacZ Forward 5'-AGT TCA CCC GTG CAC CGC-3'<br>LacZ Reverse 5'-CGC TCG GGA AGA CGT ACG-3'<br>Tau WT Forward 5'-ATG CGG TAC CTC TTT GGT GCT GTCC CTG C-3'<br>TAU WT Reverse 5'-CAG ACT GTG CTC CAC TGT G-3'
RC ::FELA	LacZ Forward 5'-AGT TCA CCC GTG CAC CGC-3'<br>LacZ Reverse 5'-CGC TCG GGA AGA CGT ACG-3'
ROSA26 AI 9	IMR9020-WT - Forward 5'-AAG GGA GCT GCA GTG GAG TA-3'<br>IMR9021-WT- Reverse 5'-CCG AAA ATC TGT GGG AAG TC-3'<br>IMR9103-MUT - Reverse 5'- GGC ATT AAA GCA GCG TAT CC3'<br>IMR9105-MUT- Forward 5'-CTG TTC CTG TAC GGC ATG G-3'
Atoh1 probe	Forward 5'-CGATTTTAGGTGACACTATAGAAATCAACGCTCTGTCGGAGTT-3'<br>Reverse 5'-CTAATACGACTCACTATAGGGACAGAGGAAGGGGATTGGAAGAG - 3'
Cited1 probe	Forward 5'-TGGGGGGCTTAAGAGCCCGG-3'<br>Reverse 5'-AGGTGAGGGGTAGGATGCAG-3
