# Supplementary material for: A medullary centre for lapping in mice
Source: Nat Commun. 2021 Nov 2;12:6307. doi: 10.1038/s41467-021-26275-y (PMC8563905; doi:10.1038/s41467-021-26275-y)
Supplement: Supplementary file 5 — Description of Additional Supplementary Files [file 41467_2021_26275_MOESM5_ESM.pdf]

Title: Supplementary Movie 1
Description: Stimulation (1000 ms) of IRt^{Phox2b}. Displayed at ¼ speed.

Title: Supplementary Movie 2
Description: Stimulation (1000 ms) of $\mathrm{Peri5}^{Atoh1}$. Displayed at ¼ speed

Title: Supplementary Movie 3
Description: Activity of IRt^{Phox2b} during a spontaneous licking bout. Displayed at ¼ speed.
