# Supplementary material for: A medullary centre for lapping in mice
Source: Nat Commun. 2021 Nov 2;12:6307. doi: 10.1038/s41467-021-26275-y (PMC8563905; doi:10.1038/s41467-021-26275-y)
Supplement: Supplementary file 6 — Reporting Summary [file 41467_2021_26275_MOESM6_ESM.pdf]

nature portfolio

Corresponding author(s): Jean-François Brunet

Last updated by author(s): Sep 6, 2021

Reporting Summary

Nature Portfolio wishes to improve the reproducibility of the work that we publish. This form provides structure for consistency and transparency in reporting. For further information on Nature Portfolio policies, see our Editorial Policies and the Editorial Policy Checklist.

Statistics

For all statistical analyses, confirm that the following items are present in the figure legend, table legend, main text, or Methods section.

n/a	Confirmed	
☐	☒	The exact sample size (*n*) for each experimental group/condition, given as a discrete number and unit of measurement
☐	☒	A statement on whether measurements were taken from distinct samples or whether the same sample was measured repeatedly
☐	☒	The statistical test(s) used AND whether they are one- or two-sided<br>*Only common tests should be described solely by name; describe more complex techniques in the Methods section.*
☒	☐	A description of all covariates tested
☒	☐	A description of any assumptions or corrections, such as tests of normality and adjustment for multiple comparisons
☐	☒	A full description of the statistical parameters including central tendency (e.g. means) or other basic estimates (e.g. regression coefficient) AND variation (e.g. standard deviation) or associated estimates of uncertainty (e.g. confidence intervals)
☐	☒	For null hypothesis testing, the test statistic (e.g. *F*, *t*, *r*) with confidence intervals, effect sizes, degrees of freedom and *P* value noted<br>*Give P values as exact values whenever suitable.*
☒	☐	For Bayesian analysis, information on the choice of priors and Markov chain Monte Carlo settings
☒	☐	For hierarchical and complex designs, identification of the appropriate level for tests and full reporting of outcomes
☒	☐	Estimates of effect sizes (e.g. Cohen's *d*, Pearson's *r*), indicating how they were calculated

Our web collection on statistics for biologists contains articles on many of the points above.

Software and code

Policy information about availability of computer code

Data collection	Elphy (https://github.com/yzerlaut/Elphy)<br>Doric Studio v5.3.3.14(Doric Lenses Inc, Canada)<br>2ndlook software v2.0.2.0 (10 Industries)<br>NDPview2+ V2.6.17 (Hamamatsu Photonics)<br>Leica Application Suite X v3.4 (Leica Microsystems)
Data analysis	Python v3.7 (Python Software Foundation)<br>Adobe Photoshop v21 (Adobe Inc)<br>Fiji v1.53 (Images)<br>CellfHelp v10.5281 (https://doi.org/10.5281/zenodo.5508650)<br>QuickNll v2.1 (https://www.nitrc.org/projects/quicknii)<br>DeepLabCut (version 2.0.7)

For manuscripts utilizing custom algorithms or software that are central to the research but not yet described in published literature, software must be made available to editors and reviewers. We strongly encourage code deposition in a community repository (e.g. GitHub). See the Nature Portfolio guidelines for submitting code & software for further information.

Data

Policy information about availability of data

All manuscripts must include a data availability statement. This statement should provide the following information, where applicable:

- Accession codes, unique identifiers, or web links for publicly available datasets
- A description of any restrictions on data availability
- For clinical datasets or third party data, please ensure that the statement adheres to our policy

'Data Availability'

The data that support the findings of this study are provided in the Source Data file. Microscopy data are available from the corresponding author upon reasonable request. Data from the Allen Brain Atlas was used in this study.

Field-specific reporting

Please select the one below that is the best fit for your research. If you are not sure, read the appropriate sections before making your selection.

☒ Life sciences ☐ Behavioural & social sciences ☐ Ecological, evolutionary & environmental sciences

For a reference copy of the document with all sections, see nature.com/documents/nr-reporting-summary-flat.pdf

Life sciences study design

All studies must disclose on these points even when the disclosure is negative.

Sample size	The sample sizes used in this study are stated in the legends of relevant figures and in the "statistics and reproducibility" statement, and are consistent with those of comparable anatomical and functional studies in the field (e.g. doi:10.1016/j.neuron.2012.11.010., doi: 10.1016/j.cub.2020.09.014., doi: 10.1038/s41586-020-03080-z., doi: 10.1523/JNEUROSCI.0839-16.2016., doi: 10.1073/pnas.2022134118.). Sample sizes were chosen to support meaningful conclusions in accordance with ethical committee requirements to limit the use of animals, whilst being adequate in magnitude, to ensure the consistency of qualitative and quantitative observation.
Data exclusions	There are no data exclusions
Replication	All anatomical and functional experiments were replicated across multiple individuals and in the case of functional experiments, across multiple trials per individual. The variability is reported as SEM or SD in the text and figures.
Randomization	This is not applicable as there were no group allocations for any of our studies. We made qualitative and quantitative assessments of the anatomical / functional data across multiple individuals of the same type for each experimental series.
Blinding	This is not applicable as there were no group allocations for any of our studies.

Reporting for specific materials, systems and methods

We require information from authors about some types of materials, experimental systems and methods used in many studies. Here, indicate whether each material, system or method listed is relevant to your study. If you are not sure if a list item applies to your research, read the appropriate section before selecting a response.

Materials & experimental systems

n/a	Involved in the study	
☐	☒	Antibodies
☒	☐	Eukaryotic cell lines
☒	☐	Palaeontology and archaeology
☐	☒	Animals and other organisms
☒	☐	Human research participants
☒	☐	Clinical data
☒	☐	Dual use research of concern

Methods

n/a	Involved in the study	
☒	☐	ChIP-seq
☒	☐	Flow cytometry
☒	☐	MRI-based neuroimaging

Antibodies

Antibodies used	Primary antibodies used were: goat anti-Phox2b (RD system AF4940, diluted 1:100), rabbit anti-peripherin (Abcam ab4666, 1:1000), guinea-pig anti-Lmx1b (Müller et al., 2002, 1:1000), goat anti-ChAT (Millipore AB144p), 1:100), chicken anti BGal (Abcam, ab9361, 1:1000), chicken anti-GFP (Aves labs, GFP-1020,1:1000), rabbit anti-GFP (Invitrogen, A11122, 1:1000), rabbit anti-Phox2b (Pattyn et

al., 1997 ,1:500), rat anti-RFP (Chromotek, 5F8, 1:1000), goat anti-CTB (List Labs, #703, 1:500). All secondary antibodies were used at 1:500 dilution: donkey anti-chicken 488 (Jackson laboratories, 703-545-155), donkey anti-chicken Cy5 (Jackson laboratories, 703-176-155), donkey anti-goat Cy5 (Jackson laboratories, 705-606-147), donkey anti-rabbit 488 (Jackson laboratories, 711-545-152) donkey anti-rabbit Cy5 (Jackson laboratories, 712-165-153), donkey anti-rat Cy3 (Jackson laboratories, 711-495-152), donkey anti-Guinea pig Cy3(Jackson laboratories 706-165-148), sheep anti-DIG antibody (Roche diagnostics, 11093274910), biotinylated goat anti-rabbit (Vector Laboratories, PK-6101), biotinylated rabbit anti-goat (Vector Laboratories, PK-4005).

Validation

Goat anti-Phox2b: Species reactivity for Human and Mouse as per the manufacturer, "PHOX2B was detected in immersion fixed IMR-32 human neuroblastoma cell line using Goat Anti-Human PHOX2B Antigen Affinity-purified Polyclonal Antibody (Catalog # AF4940) at 10 µg/mL for 3 hours at room temperature. Cells were stained using the NorthernLights™ 557-conjugated Anti-Goat IgG Secondary Antibody (red, upper panel; Catalog # NL001) and counterstained with DAPI (blue, lower panel). Specific staining was localized to nuclei and cytoplasm"

Rabbit anti-peripherin: Species reactivity for Rat and Human as per the manufacturer, "Immunocytochemistry/Immunofluorescence analysis of mixed neuron/glia cultures from newborn rat brain labelling Peripherin (green) with ab4666 and Neurofilament heavy polypeptide (red) with ab4680. Anti-Peripherin antibody (ab4666) at 1/1000 dilution + Rat spinal cord homogenate via western blot" Validated on mouse embryo by doi: 10.1242/dev.185199.

Guinea-pig anti-Lmx1b: Species reactivity for Mouse. Validated via IHC on mouse embryos by doi: 10.1016/s0896-6273(02)00689-x.

Goat anti-ChAT: Species reactivity for Human, Rat, Mouse, Monkey, Opossum, Avian, Chicken, Guinea Pig, Zebrafish as per the manufacturer, "Goat anti-ChAT (Catalogue No. AB144P) staining of organotypic slice cultures of septum from 7-day-old rat tissue maintained in culture for 8 days (IHC). Coronal section of rat neocortex stained with catalog number AB144P showing the choline acetyltransferase positive cholinergic nerve fibers and terminals. Reference: D. Anandh, K Shobha and Dr. Bindu M Kutty, Department of Neurophysiology, National Institute of Mental Health and Neurosciences, Bangalore, India.Western Blot Analysis: Representative lot data. NIH/3T3 lysate was resolved by electrophoresis, transferred to PVDF membrane and probed with anti-CHAT (1:1000 dilution). Proteins were visualized using a rabbit anti-goat secondary antibody conjugated to HRP and a chemiluminescence detection system. Validated via IHC in Mouse by doi: 10.7554/eLife.06412.

Chicken anti-BGal: Species reactivity for Mouse as per the manufacturer, "P0-adult mice were euthanized and perfused with 4% paraformaldehyde in PBS (PF). Their spinal cords were then post-fixed for 30–60 mins in 4% PF at 4°C (P0) or at room temperature (adult). Spinal cords were rinsed and cryoprotected in 20% sucrose in PBS (4°C) prior to embedding in OCT (Tissue-Tek). Immunostaining of frozen spinal sections was performed by incubating 20 µm thick sections with primary antibodies, which were then detected using species-specific secondary antibodies conjugated with Cy2, Cy3 and Cy5 or FITC. ab9361 was used at 1:1000. doi: 10.1371/journal.pone.0077928.

Chicken anti-GFP: Species reactivity for Mouse as per manufacturer, "Antibodies were analyzed by western blot analysis (1:5000 dilution) and immunohistochemistry (1:500 dilution) using transgenic mice expressing the GFP gene product. Western blots were performed using BlokHen® (Aves Labs) as the blocking reagent, and HRP-labeled goat anti-chicken antibodies (Aves Labs, Cat. #H-1004) as the detection reagent. Immunohistochemistry used tetramethyl rhodamine-labeled anti-chicken IgY." Validated via IHC in Mouse by doi:10.1523/ENEURO.0174-16.2016

Rabbit anti-GFP: Species reactivity for Rat, Fruit fly, Sheep, Zebrafish, Mouse, Human as per manufacturer, "Immunofluorescent analysis of GFP Tag was performed using H3-GFP construct transfected in HEK-293E cells. The cells were fixed with 4% paraformaldehyde for 10 minutes, permeabilized with 0.1% Triton™ X-100 for 15 minutes and blocked with 2% BSA for 1 hour at room temperature. The cells were labeled with GFP Polyclonal Antibody (Product # A-11122) at 1:200 dilution and Histone H3 Monoclonal Antibody (865R2) Product # AHO1432) at 1:200 dilution in 0.1% BSA, incubated at 4 degree celsius overnight and then labeled with Goat anti-Rabbit IgG (H+L) Highly Cross-Adsorbed Secondary Antibody, Alexa Fluor Plus 555 (Product # A32732) and Goat anti-Mouse IgM (Heavy Chain) Secondary Antibody, Alexa Fluor 647 (Product # A-21238)". "Antibody specificity was demonstrated by detection of different targets fused to GFP tag in transiently transfected lysates tested. Relative detection of GFP tag was observed across different proteins fused with GFP in H3-GFP (Lane 3-5) and p65-GFP (Lane 6). GFP-variant, YFP is also being detected in His-p65-YFP lysate (Lane 7), using Anti-GFP Polyclonal Antibody (Product # A-11122) in Western Blot. " Validated via IHC in Mouse by doi: 10.1038/s41598-020-71681-9.

Rabbit anti-Phox2b: Species reactivity for Mouse. "An antiserum was produced (Neosystem) against a BSA-coupled 15mer corresponding to the C terminus of the Phox2b protein with an added N-terminal tyrosine (YPNGAKAALVKSSMF). The antiserum was tested by ELISA. Validated via IHC in Mouse embryos by doi: 10.1242/dev.124.20.4065."

Rat anti-RFP: Species reactivity for Human and Mouse as per manufacturer, " HeLa cells stably expressing mCherry-PCNA. Primary antibody: [5F8] 1:1,000 Secondary antibody: anti-rat Alexa Fluor® 488 (IHC). Validated via IHC in Mouse C2C12 myoblasts by doi: 10.1089/hyb.2008.0031.

Goat anti-CTB: Species reactivity for Mouse. Validated via IHC in Mouse by DOI: 10.1152/ajpregu.00094.2015

Animals and other organisms

Policy information about studies involving animals; ARRIVE guidelines recommended for reporting animal research

Laboratory animals	The following transgenic mouse lines were used: Rosa FTLG, Phox2b::Flpo; Phox2b::Cre, VGlut2::Cre, Atoh1::Cre, Atoh1::FRTCre, Olig3::CreERT2 (31), FoxgliresCre, RC::FELA(60) Tau::Syp-GFP (61), RosanlslacZ and Ai9. Experiments were performed on embryos at embryonic (E) days E11.5-17.5, neonate pups at postnatal day 2-8 (P2-8) and adult (P30-56) animals of either sex. All mice were produced in a B6D2 background.
Wild animals	There were no wild animals used in the study
Field-collected samples	There were no field collected samples used in the study
Ethics oversight	All procedures were approved by the Ethical Committee CEEA-005 Charles Darwin (authorization 26763-2020022718161012) and conducted in accordance with EU Directive 2010/63/EU.

Note that full information on the approval of the study protocol must also be provided in the manuscript.
